# The Role of Nut Sensitization in Pru p 3-Sensitized Patients: A XGBoost and Generalized Linear Model Application

**DOI:** 10.3390/ijms27031223

**Published:** 2026-01-26

**Authors:** Sebastiano Gangemi, Giuseppe Caristi, Clara Alessandrello, Francesca Dimasi, Federica Nuccio, Michael Morabito, Paola L. Minciullo

**Affiliations:** 1 School and Operative Unit of Allergy and Clinical Immunology, Department of Clinical and Experimental Medicine, University Hospital of Messina, 98125 Messina, Italy; sebastiano.gangemi@unime.it (S.G.); clara.alessandrello@outlook.it (C.A.); francescadimasi5@gmail.com (F.D.); federica.nuccio01@gmail.com (F.N.); 2 Department of Economics, Messina University, Via dei Verdi, 75, 98122 Messina, Italy; gcaristi@unime.it; 3 Department of Political and Juridical Sciences, University of Messina, Piazza XX Settembre, 4, 98122 Messina, Italy; maicol.morabito@gmail.com

**Keywords:** artificial intelligence, machine learning, XGBoost model, food allergy, lipid transfer protein, Pru p 3, peach allergy, tree nut allergy, cross-reactivity, LTP syndrome

## Abstract

Sensitization to non-specific lipid transfer proteins (nsLTPs) is highly prevalent in Mediterranean countries. Pru p 3 from peach is a major allergen responsible for IgE-mediated food allergies. As a panallergen, Pru p 3 shows high sequence homology with nsLTPs from other Rosaceae fruits but also from botanically unrelated sources, including nuts and pollens, leading to extensive cross-reactivity complicating diagnosis and management. Given the worldwide prevalence of peanut and tree nut allergies, this study aimed to investigate sensitization patterns in Pru p 3-sensitized patients with tree nut allergy, using artificial intelligence (AI) to identify predictors of clinical reactivity and severity. Data from Pru p 3–sensitized patients with symptoms to peach and/or nuts were analyzed. Sensitization profiles were modeled using an XGBoost algorithm to explore associations with symptoms and severity. Patients sensitized to Pru p 3 and symptomatic for peach and nuts showed predominant sensitization to peanut and hazelnut, but AI revealed stronger associations between clinical reactivity and sensitization to hazelnut, walnut, and almond. Among patients with nut allergy and peach-asymptomatic, peanut and hazelnut sensitization were most frequent, while peach-symptomatic ones, walnut and almond sensitization predominated. Overall, walnut sensitization emerged as the main predictor of clinical severity and increasing number of sensitizations correlated with higher severity. The XGBoost algorithm identified specific allergen combinations associated with symptoms and severity, highlighting walnut sensitization as the strongest severity predictor. Machine learning approaches represent a promising tool for refining risk stratification and personalizing management in nsLTP-related food allergy.

## 1. Introduction

### 1.1. nsLTPs

Non-specific lipid transfer proteins (nsLTPs) are a widespread family of proteins in the plant kingdom and are classified within the prolamin superfamily. Despite limited sequence homology, the superfamily includes cereal seed storage prolamins (gliadins and glutenins), α-amylase inhibitors, 2S albumin seed storage proteins, and nsLTPs.

All four families exhibit immunogenic potential and are capable of triggering IgE-mediated sensitization in predisposed individuals [1,2,3].

nsLTPs possess a conserved structure composed of four α-helices, eight cysteine residues forming four disulfide bridges, and a hydrophobic cavity. This structural configuration confers high resistance to thermal processing and proteolytic degradation, including by gastrointestinal enzymes [4].

Their expression is highest in epidermal tissues, the pericarp, and the peel of plant foods, decreasing toward the inner pulp [5]. nsLTPs are also expressed in pollen from several allergenic plant families, including Compositae, Oleaceae, Urticaceae, Platanaceae, and Cannabis sativa [6].

### 1.2. Peach nsLTP: Pru p 3

Pru p 3, the major allergen of peach (*Prunus persica*), was the first nsLTP to be identified and characterized. Pru p 3 appears to be one of the most widespread allergens responsible for IgE-mediated food allergies in Mediterranean countries. Pru p 3 is a small, basic protein composed of 91 amino acids, with a molecular weight of 9.178 kDa and heat-stability and resistance to proteolytic digestion in the gastrointestinal tract. Three IgE-binding epitopes have been identified on the LTP molecule, which share sequence identities ranging from 62% to 81% with homologous regions in other fruits, including apple, apricot, plum, cherry, orange, strawberry, and grape [6].

### 1.3. Allergy to nsLTPs

The prevalence of nsLTPs sensitization appears to be influenced by climatic and environmental factors. Indeed, nsLTPs sensitization is more commonly observed in southern regions of Spain and Italy, characterized by a Mediterranean climate, as compared to the northern regions of these countries, which experience a more continental climate. However, variable rates of sensitization have also been reported in “non-endemic” countries such as Portugal, with its Atlantic climate, and in non-European nations such as China [6].

Fruits belonging to the Rosaceae family are key triggers of IgE-mediated allergic reactions in individuals sensitized to nsLTPs [7].

IgE-mediated sensitization to nsLTPs may result in a wide spectrum of clinical manifestations, ranging from contact urticaria or pruritus, to oral allergy syndrome (OAS), systemic involvement, and in some cases, life-threatening anaphylaxis [8].

Pru p 3 is considered a pan-allergen as it has a high degree of sequence homology with nsLTPs from other Rosaceae fruits, with sequence identity ranging between 88% and 97% [9]. NsLTPs share a common structural architecture, despite the sequence identity between nsLTPs from botanically unrelated species it tends to be low [10].

**Figure 1:**
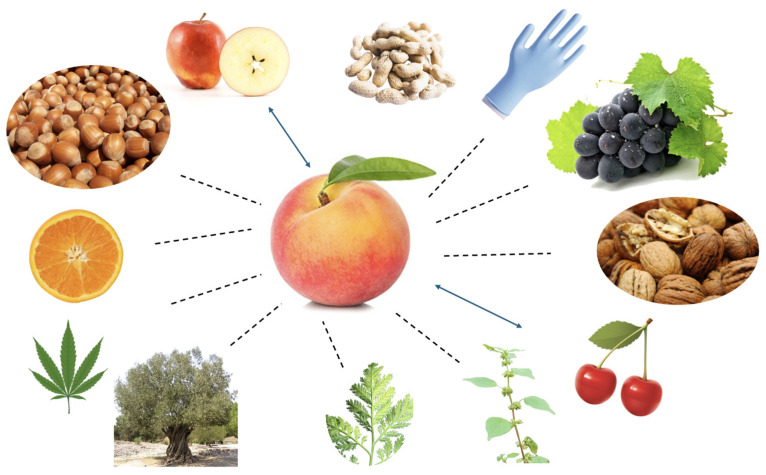
Cross-reactivity between nsLTPs belonging to different allergenic sources. Arrows link peach to Rosaceae fruits; dashes lines link peach to fruits and plants not belonging to the Rosaceae family.

These characteristics contribute to the high degree of cross-reactivity between peach and both tree nuts and pollens from various plants and trees in the context of nsLTPs allergy (Figure 1). This extensive cross-reactivity complicates both the diagnosis and management of nsLTPs-sensitized patients and has led to the conceptualization of a distinct clinical entity known as LTP syndrome.

LTP syndrome is defined as sensitization to at least two phylogenetically unrelated nsLTPs from different sources, or to more than two nsLTPs derived from the same botanical family, i.e., taxonomically related species [11].

In the specific context of nsLTPs, multiple factors contribute to the broad variability in clinical presentation [12].

For example, the presence of only mild local symptoms such as OAS, can often be explained by the patient’s individual sensitization profile, such as the co-sensitization to other allergen families, as PR-10 proteins or profilins, appears to exert a protective effect against the development of severe allergic reactions [13].

Conversely, several co-factors may worsen the clinical manifestations of nsLTPs allergy, as the ingestion of the sensitizing food on an empty stomach, concurrent intake of NSAIDs or pump proton inhibitor (PPI), alcohol consumption, menstruation, physical exercise performed shortly after ingesting the sensitizing food (food-dependent exercise-induced anaphylaxis—FDEIA).

In nsLTPs-sensitized individuals, allergic symptoms may manifest only in the presence of one or more co-factors [14].

Table 1 summarizes the protective and prognostically negative cofactors in patients sensitized to nsLTPs.

### 1.4. Cross-Reactivity Between Peach and Tree Nut Allergens

NsLTPs derived from phylogenetically distant allergenic sources exhibit varying degrees of cross-reactivity. These proteins are responsible for both allergic cross-reactions between foods from different botanical families and sensitizations to LTP-containing foods in the absence of allergic symptoms [15].

A class of foods that frequently cross-reacts with Pru p 3 includes tree nuts, namely almond, walnut, hazelnut, and peanut.

Almond belongs to the Rosaceae family. The sequence identity between peach and almond LTPs is 93.4% (85 identical amino acid residues out of 91) [16]. However, such a high sequence identity does not always correspond to a high level of clinical cross-reactivity [17].

The other nuts—walnut, hazelnut, and peanut—belong to phylogenetically distant families: Juglandaceae, Betulaceae, Leguminosae, respectively.

Various laboratory techniques, such as RAST inhibition and ELISA inhibition assays, are employed to determine whether clinical hypersensitivity to non-Rosaceae foods in patients sensitized to Pru p 3 is due to cross-reactivity between the respective nsLTPs [18,19].

Concerning hazelnut sensitization, it is known to be predominantly LTP-driven in Spain and Italy, whereas in Northern European countries it is mainly associated with birch pollen allergy [20].

Cross-reactivity between Cor a 8 (hazelnut LTP) and Pru p 3 has also been investigated using ELISA inhibition analysis, which showed partial inhibition of IgE binding to Pru p 3 by Cor a 8 [21]. A comparison of the amino acid sequences of Pru p 3 and Cor a 8 (91 residues) revealed a sequence identity of 57% [9].

It is known that sensitization is not the same as allergy, since a patient sensitized to an allergen test positive for the allergen (in vivo or in vitro) does not necessarily have symptoms. An allergic patient shows allergic symptoms after ingesting foods to which they tested positive. Therefore, among patients sensitized to food allergens, it is possible to distinguish between symptomatic patients (allergic), and asymptomatic patients (sensitized to the allergen, but tolerant to ingestion of the food involved).

### 1.5. Application of Artificial Intelligence: XGBoost in Epidemiological Modeling

In recent years, artificial intelligence (AI) algorithms, such as those based on machine learning and gradient boosting, have revolutionized numerous fields: from finance to engineering, from agriculture to medicine and epidemiology. These methods excel at managing complex, high-dimensional data with non-linear relationships, overcoming the limitations of traditional statistical models. In the medical field, they support early diagnosis, epidemic prediction, and personalized analysis by learning hidden patterns from empirical data. Their flexibility and scalability make them ideal for large-scale real-world applications.

Among these algorithms, XGBoost represents an optimal choice for this study, motivated by its proven effective learning capability and its flexibility in handling complex and high-dimensional datasets, typical features of modern epidemiological data. Compared to traditional epidemiological methods, such as classical linear or logistic regression models, XGBoost offers advantages stemming from gradient boosting-based artificial intelligence techniques, which enable modeling of nonlinear and complex relationships between variables without requiring rigid a priori specification of their functional forms [22]. As a scalable implementation of this technique, XGBoost enhances its effectiveness through advanced computational optimizations, including regularization and parallelization, making it particularly suitable for large-scale epidemiological datasets [23]. Applications in epidemiology, such as dengue outbreak forecasting or health risk modeling, demonstrate its utility in capturing nonlinear interactions in real-world contexts. This approach facilitates the identification of complex, nonlinear interaction patterns that are difficult to detect using traditional epidemiological models, which often rely on parametric and linearity assumptions [22]. Furthermore, gradient boosting-based machine learning methods like XGBoost improve predictive performance through an iterative optimization process that progressively reduces residual errors. Empirical evidence of these advantages emerges from large-scale clinical–epidemiological applications, as shown in the study by Cao et al. (2023), which developed and validated an XGBoost-based survival model outperforming traditional models [24]. XGBoost also enables effective management of complexity via regularization and overfitting control, which are essential for epidemiological data often characterized by noise and high variability [23]. The second-order optimization procedures inherent to the XGBoost algorithm enhance model robustness and stability, overcoming limitations of conventional statistical methods. From a computational perspective, XGBoost’s optimizations such as parallelization and efficient memory management allow rapid analysis of large datasets without compromising accuracy or predictive quality [23]. Finally, the artificial intelligence-based approach reduces the need for manual variable selection and transformation, automating model building and making the method more scalable and applicable across diverse data types and epidemiological contexts. This positions XGBoost as particularly well-suited to complex problems where traditional models prove less effective or overly restrictive.

### 1.6. Aim of the Study

The prevalence of peanut and nut allergies worldwide is high, but the pattern of sensitization varies greatly in different geographical areas depending on the allergen family involved. Given the high incidence of nsLTP allergy and the predominant prevalence of peach and nuts in triggering allergic symptoms in our latitude (Mediterranean basin), the aim of this study is to analyze the clinical characteristics of patients simultaneous sensitized to Pru p 3 and nuts, distinguishing patients who tolerate the ingestion of peach or nuts from patients who presented allergic symptoms to one or both the foods involved.

Specifically, the following objectives will be assessed:

(1) To identify which specific nuts were responsible for adverse allergic reactions both in peach sensitized or allergic patients.
(2) To determine the proportion and characteristics of patients sensitized to peach LTP but asymptomatic compared to those with allergic symptoms.
(3) To assess whether the number or type of sensitization correlates with clinical severity of the allergic reactions.

## 2. Results

### 2.1. Database Results

**Figure 2:**
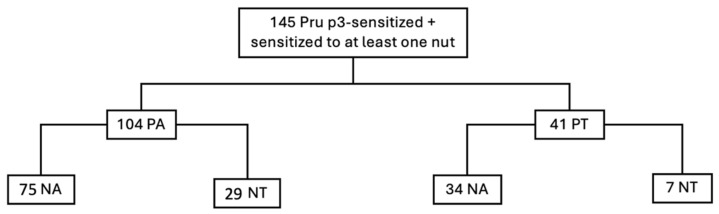
Graphical representation of the study cohort. Legend: PA: Peach allergic; PT: Peach tolerant; NA: Nuts allergic; NT: Nuts tolerant.

Among the 145 patients simultaneously sensitized to peach and nuts, 104 patients experienced symptoms following contact or ingestion of peach (peach allergic patients), 41 patients reported no allergic symptoms either from contact with or ingestion of peach or peach skin peel (peach tolerant patients) (Figure 2).

In this group of 41 patients asymptomatic for peach, 7 were also asymptomatic for nuts and came to the allergy clinic for reported adverse reactions to other foods. Maybe the reactions reported were caused by food contamination with nuts.

### 2.2. The AI Results

The results were analyzed focusing on three main objectives:

(1) Identify which specific types of nuts are responsible for allergic reactions. Among the 145 patients, 109 (75.17%) were sensitized to Pru p 3 (both symptomatic and asymptomatic for peach) and had symptoms related to nuts; we therefore investigated which nut family was most responsible in symptomatic patients. In the 104 patients (71.73%) symptomatic for peach, we studied which type of nuts they were sensitized to, regardless of the development of clinical symptoms.
(2) Evaluate the proportion and characteristics of patients sensitized to Pru p 3 but asymptomatic compared to those with clinical symptoms. In particular, among the 41 patients asymptomatic for peach, 34 subjects showed symptoms to nuts; the other 7 patients were excluded because they were asymptomatic for both peach and nuts. We investigated whether there were common characteristics in these patients and which nut family predominantly induced symptoms.
(3) Explore whether the number or type of sensitizations correlated with the clinical severity of allergic reactions in the entire population. We considered 109 patients symptomatic for nuts: 75 had symptoms with both (peach and nuts) and 34 had symptoms only with nuts. We also want to analyze in this latter subgroup the correlation between the severity of symptoms and the number and type of nuts sensitivities. We classified the severity of symptoms for both peach and nuts with a numerical value from 0 to 4, considering the following:
  ➔ 0: no symptoms;
  ➔ 1: contact symptoms (mild);
  ➔ 2: ingestion symptoms involving only the skin or gastrointestinal tract (mild–moderate);
  ➔ 3 ingestion symptoms involving the respiratory system (moderate);
  ➔ 4: ingestion symptoms involving 2 or more organs/systems (anaphylaxis) (severe).

#### 2.2.1. AI Results for the First Aim

**Identify which specific types of nuts are responsible for allergic reactions.** Among the 109 patients sensitized to Pru p 3 and symptomatic for nuts, positivity to peanuts and hazelnuts was prevalent. As shown in Table 2, peanut was identified in 87.2% of patients, followed by hazelnut (83.5%), almond (58.7%), and walnut (39.4%).

A similar pattern is observed in 104 symptomatic patients for peach and sensitized for nuts (regardless the development of clinical symptoms), where the most prevalent nuts are still peanuts (85.6%) and hazelnuts (82.7%) (Table 3). The frequencies of almonds (51.9%) and walnuts (36.5%) remain at lower levels. The figure showing these results can be found in the Supplementary File (Figure S1).

To reinforce the analysis, we applied XGBoost modelling to the cohort of patients exhibiting symptoms to tree nuts. Gain, Cover, and Frequency denote the three standard feature importance metrics yielded by XGBoost: Gain quantifies the average improvement in error reduction attributable to the feature’s splits; Cover reflects the proportion of observations influenced by those splits; and Frequency captures the relative frequency of the variable’s utilization across the ensemble trees. In Table 4, these metrics confirm the dominant role of HAZELNUT_NUM (Gain 0.34, Cover 0.46, Frequency 0.30), WALNUT_NUM (Gain 0.31, Cover 0.21, Frequency 0.27), and ALMOND_NUM (Gain 0.30, Cover 0.26, Frequency 0.38) as primary predictors of post-ingestion clinical symptoms, whereas PEANUT_NUM exhibits markedly lower predictive importance (Gain 0.05, Cover 0.06, Frequency 0.04), despite representing the most frequent sensitization (Table 4).

Table 5 integrates the percentage of positive concordance (positive test + symptom present), which clinically validates the algorithmic predictivity: HAZELNUT_NUM 96.6%, WALNUT_NUM 96.2%, ALMOND_NUM 95.2%, PEANUT_NUM 94.4%. These high percentages (>94%) confirm the strong association between skin positivity and clinical outcome, but the differential predictive importance highlights the unique value of Betulaceae and Juglandaceae in distinguishing between non-specific sensitization and actual symptomatic risk. The figure showing these results is available in the Supplementary Materials (Figure S2).

**Figure 3:**
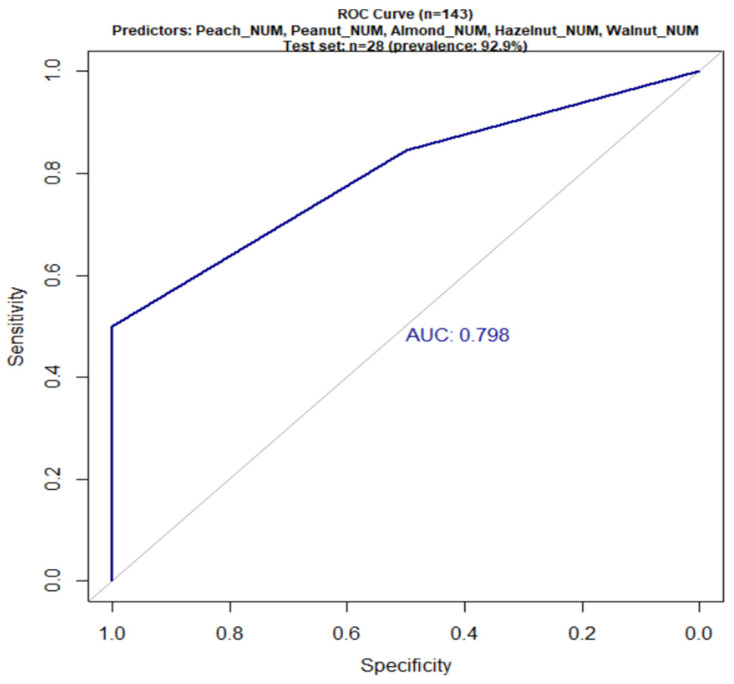
ROC curve of the XGBoost model (n = 143 patients with complete data; training n = 115, test n = 28). Predictors: Peach_Num, Peanut_Num, Almond_Num, Hazelnut_Num, Walnut_Num (0–3 scale). AUC = 0.798.

To complete the analysis of the first objective, an XGBoost model was developed and validated to predict food allergy, defined as positive skin prick test concomitant with clinical symptoms for peach or tree nuts in Pru p 3-sensitized patients (total sample n = 145). The predictors comprised numerical SPT results for five food allergens: peach (PEACH_NUM), peanut (PEANUT_NUM), almond (ALMOND_NUM), hazelnut (HAZELNUT_NUM), and walnut (WALNUT_NUM). The binary target (SYMPTOM_PRESENT = 1) identified patients with a positive test and clinical symptoms for peach or positive test to ≥1 tree nut and symptoms for tree nuts. After excluding 2 observations with missing data, the model was trained on 115 patients (80% of n = 143 complete cases) and validated on an independent test set of 28 patients (20%; allergy prevalence 92.9%), achieving 92.9% accuracy and an AUC of 0.798, as shown in Figure 3.

Specificity reached 100%, while balanced accuracy settled around 50%, a result consistent with the marked class imbalance and the prevalence of symptomatic subjects of approximately 7% in the test set, as reported in Table 6. In this context, sensitivity was 0, meaning no symptomatic subjects were correctly identified as such, despite a negative predictive value of about 0.93, which reflects the high proportion of true non-symptomatic cases among those classified as negative. This combination of maximum specificity, null sensitivity, and balanced accuracy around 50% indicates that the model effectively discriminates the non-symptomatic class but fails to distinguish the few symptomatic subjects from non-symptomatic ones, yielding overall modest classification performance in the presence of strong class imbalance.

A k-fold (5-fold) cross-validation confirmed the stability of performance, identifying an optimal model with a ROC of approximately 0.83, while a bootstrapping analysis with 100 resamples yielded a mean AUC of 0.772 and a standard deviation of 0.105, supporting the model’s robustness against sample variability.

**Figure 4:**
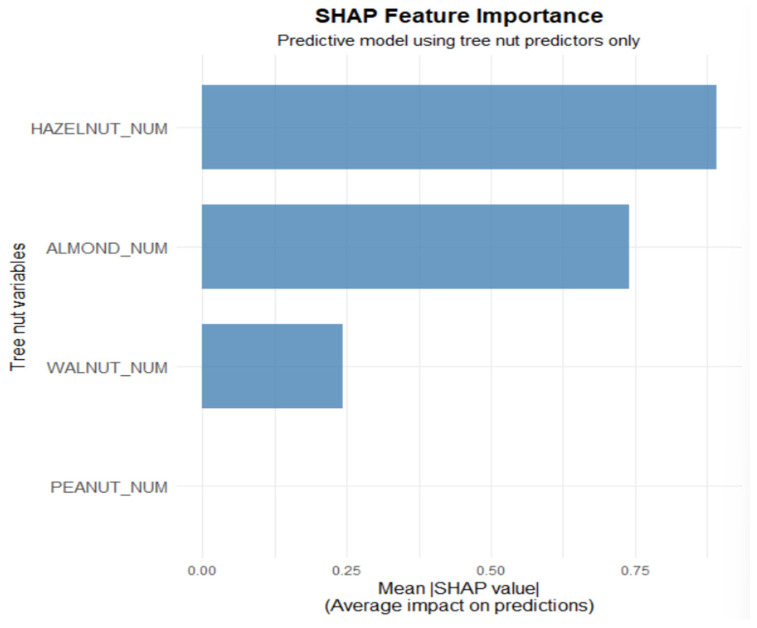
Mean importance of SHAP values for each allergen.

In addition to the global performance metrics, an interpretability analysis based on SHAP values was carried out to clarify the contribution of individual allergens to the probability of developing clinical symptoms. In this framework, HAZELNUT_NUM and ALMOND_NUM emerged as the predictors with the largest mean absolute SHAP values and therefore as the most influential factors in the model’s decisions (Figure 4).

**Figure 5:**
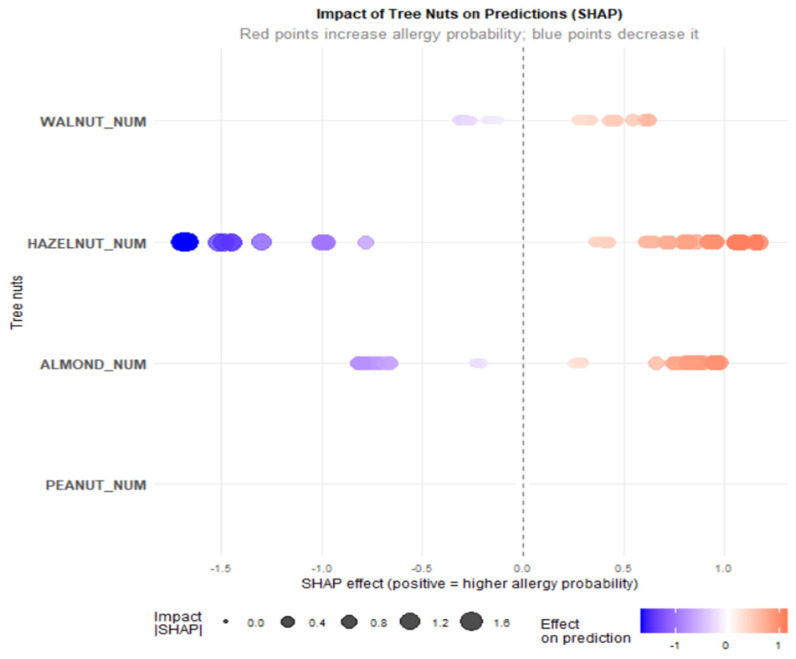
SHAP beeswarm plot for allergen.

**Figure 6:**
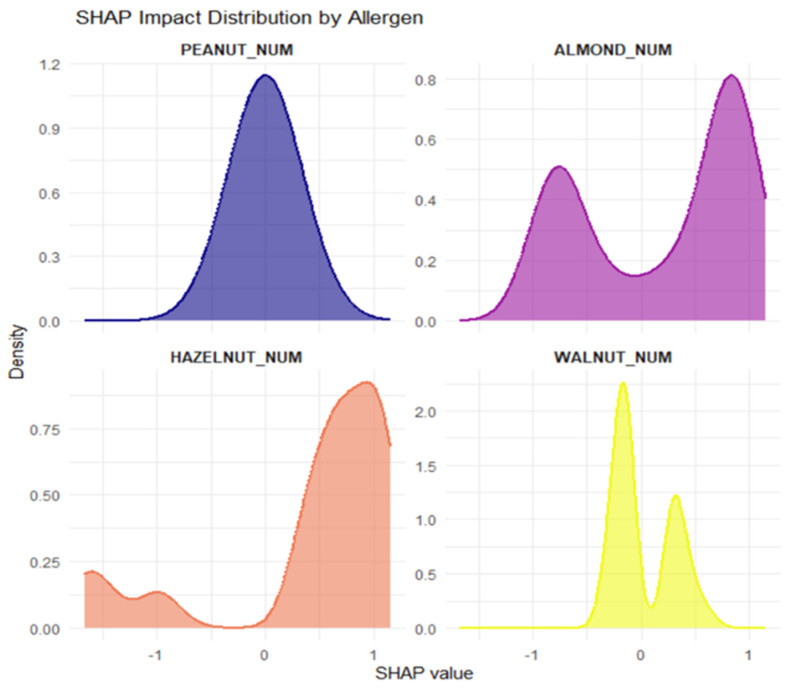
SHAP impact distribution by allergen.

In the point-wise SHAP visualizations (Figure 5), HAZELNUT_NUM shows contributions distributed on both the increasing and decreasing sides of allergy probability. This indicates that hazelnut sensitization does not operate as a strictly monotonic predictor but rather as a modulating one: for some patients, specific combinations of hazelnut sensitization with other allergens may increase the likelihood of symptoms, whereas in other contexts (for example, in the presence of different sensitization profiles), the same predictor may be associated with a lower estimated probability of symptoms. In terms of SHAP values, this is reflected in the coexistence of positive values (pushing predictions towards symptom development) and negative values (pushing them towards absence of symptoms) for HAZELNUT_NUM, supporting its role as a clinically relevant but context-dependent marker. By contrast, PEACH_NUM, although included in the model’s predictor set, represents a baseline condition that is almost uniformly shared across the cohort (given Pru p 3 positivity as an inclusion criterion) and is therefore not displayed in Figure 5 and Figure 6, while remaining implicitly accounted for in model construction. In SHAP terms, PEACH_NUM shows mean values close to zero and a distribution tightly concentrated around zero, indicating a negligible contribution to distinguishing symptomatic from asymptomatic patients compared with the other tree nut allergens. Clinically, this implies that Pru p 3 test positivity, although highly prevalent, does not add discriminative power in explaining why some patients develop symptoms whereas others remain asymptomatic, in contrast to sensitizations to hazelnut and almond, which emerge as more specific markers of clinical risk.

Analysis of demographic characteristics revealed no significant correlation between age or sex and the presence of allergic symptoms. This suggests that allergic profiles are not influenced by demographic variables in this cohort.

#### 2.2.2. AI Results for the Second Aim

**Evaluate the characteristics of patients sensitized to Pru p 3 but asymptomatic for peach.** Among the 41 patients (28.3%) asymptomatic for peach, 34 had symptoms related to tree nuts. This is a clinically interesting subgroup due to their atypical response, given that peach is usually the first food to trigger symptoms in individuals sensitized to Pru p 3. In this subgroup, peanuts and hazelnuts are again confirmed as the most common positive tested allergens (82.4% each), followed by almonds (73.5%) (Table 7). Walnut positivity is more limited (38.2%).

Comparison with the group of symptomatic patients for peach (Table 8) highlights that sensitivity to almonds and walnuts plays a more significant predictive role in determining the presence of clinical symptoms in this subgroup. Conversely, although peanuts are very frequently positive in absolute terms, their relative importance in the predictive model is significantly lower.

The figure that confirms this trend, suggesting that almonds and walnuts are allergens with greater discriminatory power for the presence of peach-related symptoms is present in the Supplementary File (Figure S3).

For the second analysis objective, specifically pertaining to peach-related symptoms, validation yielded a sensitivity of 85.7%, specificity of 14.3%, accuracy of 67.9%, and AUC of 0.517, indicating moderate performance for this more specific endpoint (Table 9).

These results indicate that, although peanut is a common sensitizer, its ability to predict clinical symptoms is less marked than that of almonds and walnuts.

To complete the second objective, the distribution of Pru p 3-positive patients was evaluated based on peach symptoms. Out of 145 sensitized patients, 104 (71.7%) were symptomatic and 41 (28.3%) were asymptomatic (Table 10).

This data highlights that, even with a positive test for peach, almost one third of patients do not develop clinical symptoms for peach, neither by ingestion nor by contact. This asymptomatic subgroup is particularly interesting, especially given that, in many cases, it still presents clinical symptoms towards other nuts, as shown above.

No correlations were found between age or sex and specific peach symptoms, indicating that peach-specific sensitization and symptomology are phenomena independent of patients’ demographic characteristics.

#### 2.2.3. AI Results for the Third Aim

**Correlation between sensitization profile and clinical severity of allergic reactions.** A sample of 109 patients with relevant clinical symptoms was analyzed. Patients asymptomatic for nuts were excluded to focus on a homogeneous group: 75 patients had symptoms related to both peach and nuts, and 34 patients had symptoms limited to nuts. Table 11 shows the results of an XGBoost predictive modelidentifying the allergens most strongly associated with the clinical severity of reactions, highlighting walnuts are the main predictor of severity, followed by peanuts (94 positive tests), hazelnuts (89 positive tests), and almonds (63 positive tests).

The figure that supports this result and clearly showing the relative importance of each allergen in predicting clinical severity is present in the Supplementary File (Figure S4). The trend illustrated in the figure shows how the influence of walnuts clearly exceeds that of other allergens, contributing more significantly to the risk profile.

This hierarchy is confirmed by Table 12, which explicitly maps positive test results for individual tree nuts to clinical severity scores (0–4), demonstrating that 100% of patients with positive tests exhibit severity ≥ 2 (no mild cases): peanuts show 94 positive tests with 78% at severity 2 and 22% at severity 3–4, hazelnuts 89 positive tests with 73% at severity 2 and 27% at severity 3–4, almonds 63 positive tests with 73% at severity 2 and 27% at severity 3–4, and walnuts 43 positive tests with 70% at severity 2 and 30% at severity 3–4. This distribution suggests that sensitization to these allergenic sources, particularly walnuts, is strongly correlated with moderate-to-severe clinical manifestations, validating the predictive importance derived from the XGBoost model.

**Figure 7:**
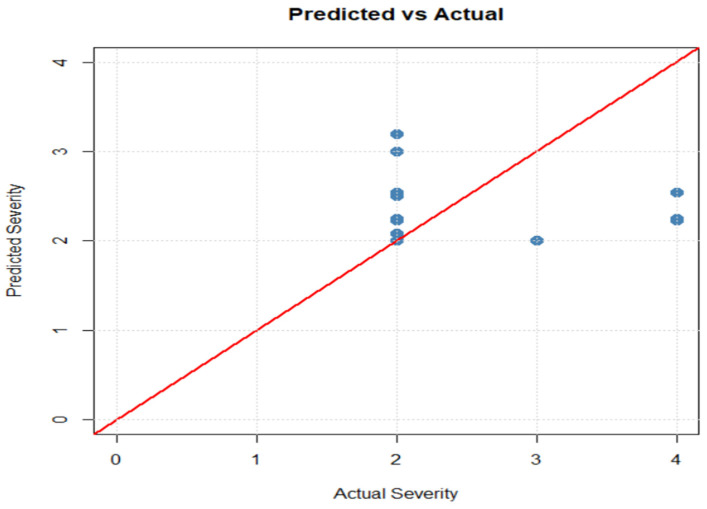
Scatter plot of predictions versus actual values of clinical symptom severity.

The regression model for symptom severity was validated using a training/test split, yielding metrics of RMSE = 0.790926, $R^{2}=0.001920$, and MAE = 0.558076. The scatter plot of actual versus predicted values reveals predictions clustered near zero, confirming limited predictive capacity for this continuous outcome, consistent with the relative importance of allergens from the XGBoost analysis (WALNUT_NUM dominant with Gain = 0.335) (Figure 7).

**Figure 8:**
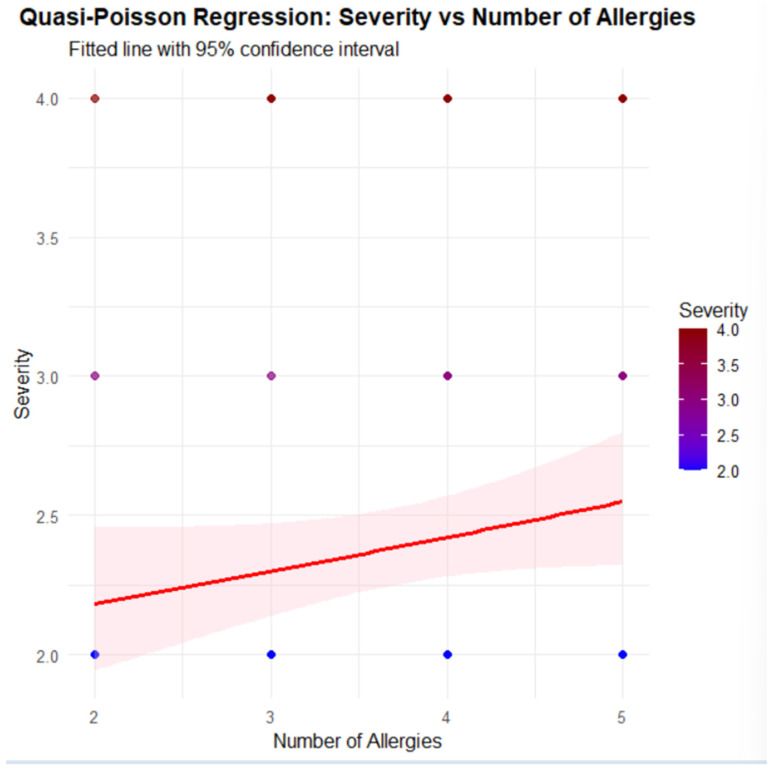
Relationship between the number of positive food allergens and clinical severity (quasi-Poisson GLM model).

At the same time, in the generalized linear regression model with a quasi-Poisson distribution, the total number of positive food allergens shows a positive coefficient (β = 0.05149, *p* = 0.0879), indicating a trend toward increased clinical severity with increasing sensitizations, although this association does not reach conventional statistical significance; this trend is consistent with that depicted in Figure 8, where the fitted regression line displays a slight positive slope and the width of the 95% confidence interval reflects the uncertainty surrounding the estimated effect. In this context, Table 13 further elucidates the role of specific allergens by comparing the frequency of sensitization between two distinct clinical groups (patients symptomatic to both foods, peach and nuts, and patients symptomatic exclusively to nuts) and shows that sensitizations to walnuts and almonds are predominantly concentrated in the group symptomatic to both foods, which exhibits the most severe clinical profile; therefore, although the overall association between the total number of allergens and severity is only suggestive, the presence of sensitization to walnuts and almonds appears to be mainly characteristic of patients with a more severe clinical phenotype, thereby pointing to a specific link between these allergens and greater symptom severity.

This trend is also depicted in Figure 8, where the fitted regression line reveals a slight positive slope in severity as the number of food allergies (NUM_ALLERGIES) increases, along with the corresponding 95% confidence interval.

## 3. Discussion

IgE-mediated reactivity to nsLTPs is the main cause of primary food allergy in adults in Mediterranean countries, with a prevalence of 9.5% reactivity to Pru p 3 observed in Italy [25]. nsLTP stability to heat and digestion by digestive enzymes makes nsLTP an allergen capable of inducing severe allergic reactions, which can even lead to anaphylaxis [26].

Pru p 3 is considered the most likely primary sensitizer to the nsLTP family and cross-reaction between peach LTP and nuts in frequent.

In our study, which covers a seven-year period between 2018 and 2024, 145 patients were identified who were simultaneously sensitized to peach and nuts. In this patient sample, we analyzed various aspects of sensitization to nsLTPs derived from Rosaceae and non-Rosaceae.

### 3.1. Aim 1: Identify Which Specific Types of Nuts Are Responsible for Allergic Reactions

From the AI analysis, among the 109 patients sensitized to Pru p 3 and simultaneously symptomatic for at least one nut, peanuts and hazelnuts were found to be positive more frequently with a percentage of 87.2% and 83.5%, respectively, followed by almond and walnut.

Peanuts cause allergy symptoms in both children and adults, with a high incidence worldwide. However, depending on the geographical region, the allergen responsible for adverse reactions to peanuts could differ. In fact, peanuts contain molecular allergens such as seed storage proteins (Ara h 1, Ara h 2, Ara h 3, Ara h 6, Ara h 7), profilin (Ara h 5), PR-10 (Ara h 8), defensins (Ara h 12, Ara h 13), oleosins (Ara h 10, Ara h 11, Ara h 14, Ara h 15), and LTP (Ara h 9) [27].

Hazelnut belongs to the Betulaceae family, and hazelnut allergy is one of the more common food allergies in Europe, with a different pattern of sensitization. Hazelnut also contains various molecular allergens, such as Bet v1-homologue (Cor a 1), profilin (Cor a 2), nsLTP (Cor a 8), seed storage proteins (Cor a 9, Cor a 11 and Cor a 14), and oleosins (Cor a 12 and Cor a 13) [28].

Almond is a Rosaceae food and contains different molecular allergens, including PR-10, LTP, profilins, and seed storage proteins. However, molecular allergens from almonds are not commonly available for study through specific IgE testing or multiplex panels [29].

Walnut belongs to Juglandaceae family and among its molecular allergens there are seed storage proteins (Jug r 1, Jug r 2, Jug r 4, Jug r 6), nsLTPs (Jug r 3 and Jug r 8), PR-10 (Jug r 5), and profilin (Jug r 7) [30].

The presence of nsLTP in peanuts suggests a certain degree of cross-reactivity between peanuts and peach [15]. Asero et al. studied the prevalence of sensitization to nuts and peanuts in a group of patients monosensitized to LTP with a clinical history of allergic reactions following the ingestion of Rosaceae fruits. All patients had positive SPT for hazelnuts, followed by peanuts and walnuts, supporting the hypothesis of strong cross-reactivity between nsLTPs contained in Rosaceae and nuts. However, their results showed a higher prevalence of almond sensitization (80%) respect to our sample [19].

The cross-reactivity between nsLTPs from different allergenic sources has been tested with various laboratory tests, such as RAST inhibition or ELISA inhibition tests. The first test was performed in a group of patients with significant IgE reactivity to walnut and/or peanut. Sera pre-absorbed with peach LTP showed complete inhibition of IgE reactivity with extracts of both walnut and peanut [19].

In a multicenter study by Romano et al. on the frequency of peanut allergy in patients sensitized to peach LTP, the patient cohort was identified through the positive SPT for peach LTP. They also searched for specific IgE for Pru p 3. Among 114 adult patients monosensitized to LTP, more than half (65%) had positive SPT for peanut, as in our case. Moreover, in vitro IgE inhibition tests confirmed that peanut LTP strongly cross-reacts with Pru p 3 [31].

An observational Spanish study aimed to assess the prevalence of sensitization to different foods in patients sensitized to peach LTP, reporting reactions after ingesting LTP-containing foods.

The authors found that the most frequently positive nuts were hazelnuts (74.22%), followed by peanuts (73.54%), and walnuts (70.98%). Almonds tested positive in 38.74% of cases [17].

Our prevalence results in the group of patients symptomatic for peach and simultaneously sensitized to at least one nut are consistent with those of the latter study: SPTs show a higher prevalence of positivity for hazelnuts and peanuts. However, compared to our results, the prevalence of walnut sensitization was high (>50%) and higher than almond sensitization. In our patients, walnut sensitization was present in less than half of the cases (Table 3).

This difference could be explained by the various sensitization patterns present in the same geographical area (the Mediterranean basin). Some studies demonstrated that the pattern of sensitization to walnuts appears to be mixed, with simultaneous sensitization to both nsLTPs and PR-10 in areas with great presence of birch trees [20,32,33].

Therefore, geographical influence and cross-reactivity with pollen could explain these different results, despite both studies being conducted in the Mediterranean basin.

In our cohort of LTP-sensitized patients, the high percentage of hazelnut sensitization would seem to reflect the data reported in the literature, according to which hazelnut allergy in the Mediterranean regions is a phenomenon determined by nsLTPs. However, in a multicenter study the sensitization pattern of patients sensitized to hazelnuts showed a mixed pattern with prevalent sensitization to PR-10, profilin, and nsLTPs [28].

It would also appear that hazelnut sensitization in adults is more frequently driven by sensitization to PR-10, compared to children. Children are more frequently sensitized to hazelnut seed storage proteins, but school-aged children living in Mediterranean countries who have OAS to hazelnuts have been found to be simultaneously sensitized to nsLTP and PR-10 [34].

With regard to the prevalence of clinical allergy to nuts, the XGBoost model showed that sensitization to hazelnuts, walnuts, and almonds is more frequently accompanied by the onset of symptoms. Peanuts, on the other hand, despite being the nuts that most often test positive, are less likely to cause symptoms. These results are consistent with the results of Asero et al., according to which the prevalence is 65% and 55% for hazelnuts and walnuts, respectively. The prevalence of clinical symptoms after peanut ingestion in peanut-sensitized patients was 40% [19]. In the study by Romano et al. [31], more than half of the patients allergic to nsLTP showed sensitization to peanuts but symptoms occurred in only about one-third of patients sensitized to nsLTPs, as demonstrated in other studies by Asero [18,35]. Romano et al. concluded that SPTs with commercial peanut extract have excellent negative predictive value, which can be very useful in clinical practice, but poor positive predictive value [31].

In light of the high degree of cross-reactivity between Pru p 3 and Ara h 9, also demonstrated by in vitro IgE inhibition and ELISA studies, we can assume that the high rate of peanut sensitization in LTP-sensitized patients is in most cases due to cross-reactivity and not to true peanut allergy. Moreover, geographical differences, age and climate change may explain the different patterns of differentiation to the same allergenic source.

### 3.2. Aim 2: Evaluate the Proportion and Characteristics of Asymptomatic Patients Sensitized to Pru p 3 Compared to Those with Clinical Symptoms

In LTP syndrome, peach is often the food responsible for the initial onset of allergic symptoms upon ingestion or contact.

However, sensitization does not necessarily correspond to clinical allergy. Although such cases appears to be relatively uncommon [6], our data confirm this possibility: in fact, nearly one-third of the subjects sensitized to Pru p 3 (41 patients) reported no allergic symptoms following contact with peel peach or ingestion of the fruit.

Among the 41 peach-tolerant patients, 34 experienced adverse reactions—ranging from mucocutaneous or respiratory involvement to anaphylaxis—after consuming at least one type of nut. Within this subgroup, peanut and hazelnut were the most frequently found positive, each exceeding 80% of cases, followed by almonds and, less commonly, walnuts (Table 7).

Based on these findings, we hypothesize that in specific patient clusters, the primary sensitizer may derive from a nsLTP source other than peach, possibly from a non-Rosaceae food or, alternatively, from an inhalant, such as Art v 3 (mugwort), Ole e 7 (olive), Par j 2 (pellitory), Can s 3 (Cannabis sativa) [36,37,38,39,40,41]. The geographical environment, and thus exposure to different allergenic sources, could influence the sensitization pattern driving the allergic reaction [42].

In our cohort, 34 patients sensitized to peach LTP extract developed symptoms primarily after nut ingestion with sensitization mainly to peanut, followed by hazelnut and, less frequently, to almond and walnut. A clinical variety of symptoms was associated. In light of these results, we assume that hazelnut or peanut could be the primary sensitizer in this population. In fact, these patients still eat and tolerate peach, further supporting this hypothesis.

Despite the high prevalence of peanut sensitization in asymptomatic patients for peach, less than 50% develop symptoms after ingesting peanuts. Therefore, it is likely that peanut sensitization is due to cross-reactivity rather than true allergy. In line with this hypothesis, there are no data in the literature suggesting peanut nsLTP may be a primary peanut sensitizer. Primary peanut allergy is usually caused by seed storage proteins [31]. Furthermore, specific IgE levels for Ara h 9 or peanuts are similar in both peanut-tolerant and peanut-allergic patients, supporting the fact that sensitization to an allergen is not the same as allergy [43].

In Europe, hazelnuts are the most common tree nut responsible for allergic sensitization [44,45], with a rate ranging from 17% to 100% across European countries [46]. The “Pronuts” study, conducted in London, Geneva, and Valencia, identified hazelnut allergy in 32% of tree nut–allergic patients [47]. In Italy, the estimated prevalence of hazelnut allergy is approximately 0.2% of the general population [48], a rate that may be related to the country’s high consumption levels.

In our cluster of 34 patients, hazelnut is the second most frequently positive nut. Unlike peanuts, there are data in the literature in which hazelnut LTP was proposed as a primary sensitizer in a group of children from Northern Europe. In this geographic area, the sensitization to tree nuts is more frequently driven by birch-pollen and linked to PR-10, but the patients showed sensitization to Cor a 8 only without Pru p 3 sensitization [49]. Similarly, in our group of patients sensitized to peach LTP, but asymptomatic for peach, we might think that LTP sensitization was driven by Cor a 8. However, this observation is merely speculative.

Indeed, although Pru p 3 is known to dominate the immunological response in patients with LTP syndrome, it could be hypothesized that in some patient clusters, the primary sensitizer is the nsLTP from a food other than peach. The geographical area in which the patient lives could play a role in the different modes of LTP sensitization. Further studies are required to confirm this hypothesis.

The subgroup of 104 patients symptomatic for peach showed a higher frequency of sensitization to almond and walnut (Table 8) using the XGBoost model application.

Although peanut sensitization was the most prevalent overall in our sample, our AI-based analysis revealed that almond and walnut sensitization occurred more frequently among patients symptomatic for peach compared with asymptomatic individuals. This finding might suggest that sensitization to almond and walnut may have a predictive value for the presence of clinical symptoms in Pru p 3-sensitized patients (Table 8), whereas peanut sensitization appears to play a marginal predictive role.

In light of these findings, our results can reflect a higher degree of sequence homology between Pru p 3 and almond, a Rosacea food, and walnut, even though it belongs to a different taxonomic group [19]. These observations suggest that, in patients symptomatic for peach, IgE binding may occur with higher receptor specificity toward epitopes shared by Pru p 3, almond LTP, and Jug r 3. This may indicate a primary sensitization to Pru p 3, which drives cross-reactivity to homologous LTPs in almond and walnut. Conversely, in asymptomatic individuals, other factors may reduce the reactivity of Pru p 3-specific IgE despite sensitization, possibly because nsLTP-specific IgE from non-Rosaceae foods such as hazelnuts or peanuts exhibit different binding accessibility to Pru p 3 epitopes.

### 3.3. Aim 3: Explore Whether the Number or Type of Sensitizations Correlated with the Clinical Severity of Allergic Reactions in the Entire Population

The predictive model based on the XGBoost algorithm revealed that among the 109 patients analyzed—75 symptomatic for both peach and tree nuts, and 34 symptomatic only to tree nuts—walnut emerged as the main predictor of clinical severity, followed by peanut, hazelnut, and almond.

This finding suggests that sensitization to nuts, particularly to walnuts, may serve as important molecular markers for identifying patients at higher risk of systemic reactions.

Although it is well established in the literature that Pru p 3 and Ara h 9 act as risk markers for anaphylaxis or systemic reactions [50], our data identifying walnuts as a potential predictor of clinical severity in LTP syndrome. This novel knowledge may reflect the high structural similarity and cross-reactivity between Jug r 3 and other LTPs, which could amplify the immune response in polysensitized individuals. The identification of Jug r 3 as the main factor of clinical severity expands the current insights into LTP syndrome, going beyond the traditionally dominant role of Pru p 3.

Another study demonstrated that nsLTP polysensitization can be associated with more severe clinical phenotypes and identified Jug r 3 as the second most common cause of anaphylaxis after Pru p 3 [51]. Our findings reinforce this notion, highlighting that the coexistence of multiple nsLTPs sensitizations—especially involving both peach and nut components—could synergistically increase clinical risk.

In our cohort, peanuts are the second indicator of clinical severity. Since peanut sensitization—and in some cases peanut allergy —is commonly observed among patients sensitized to nsLTPs, as confirmed both by our data and by previous studies [31], it is crucial to consider this allergen as a potential cofactor contributing to increased clinical risk.

Another study showed that walnuts and peanuts were the main cause of anaphylaxis in patients allergic to nsLTP [17].

Our findings also confirmed previous evidence that sensitization to more than five nsLTPs is associated with a higher risk of severe systemic reactions [25]. The GLM analysis demonstrated a positive trend between the number of allergens to which a patient is sensitized and the degree of clinical severity, suggesting that IgE-mediated sensitization to multiple allergens may reflect a more reactive or dysregulated immune profile.

Finally, comparing sensitization frequency between the two clinical groups (symptomatic to both peach and nut vs. symptomatic to nut only), greater sensitization to walnuts and almonds was observed predominantly in the first group. This finding supports the hypothesis that these sensitizations contribute to a more complex and severe clinical phenotype. From a clinical perspective, these data, appropriately revised and supported by laboratory diagnostics and a larger case series, may provide useful information for risk stratification in patients with LTP syndrome and guide more tailored management approaches, highlighting the need for a comprehensive molecular profile in individuals with multiple food allergies.

Future studies will be essential to confirm the predictive value of walnut and other nuts as biomarkers of risk for systemic allergic manifestations in patients sensitized to nsLTPs.

## 4. Material and Methods

### 4.1. Patients

This study is observational, spontaneous, and retrospective. It was conducted at the Clinical Unit of Allergology and Clinical Immunology of the University Hospital “G. Martino” in Messina, Italy.

The clinical sample consisted of 145 adult patients (91 females and 54 males) with an average age of 35 [range 18–73], who attended outpatient consultations at our Unit between January 2018 and December 2024, simultaneously sensitized to Pru p 3 and to at least one type of tree nut, including walnut, hazelnut, almond, or peanut.

The diagnosis of allergy to nsLTPs was established through clinical history collection and the execution of skin prick tests (SPTs) using standardized allergenic extracts and/or quantification of specific IgE antibodies to whole or molecular allergens. A subset of patients underwent third-level diagnostic testing (Component-Resolved Diagnostics, CRD), such as the ALEX test or ISAC test. For the patients tested via SPT with standardized peach extract, the extract used was “Peach LTP” (Lofarma s.p.a., Milan, Italy).

Patients not simultaneously sensitized to both peach and at least one tree nut were excluded from the study.

All personal and clinical data of the patients involved were processed in accordance with current privacy protection regulations, following the standard procedures already established at the University Hospital “G. Martino” in Messina. The work has been approved by the local bioethics Committee (protocol number 04/25 of 11 February 2025).

A limitation of this study is the inability to perform molecular diagnostics with specific IgE quantification for molecular allergens in all patients. Consequently, potential protective factors or prognostically unfavorable markers could not be identified or evaluated.

### 4.2. The AI Analysis

#### 4.2.1. XGBoost Model (Binary Classification and Regression)

The XGBoost algorithm represents one of the most powerful and widely used implementations of boosting methods, capable of achieving high performance in many supervised learning problems. Its basic structure applies to both binary classification and regression tasks, differing mainly in the loss function used and in how predictions are interpreted. In the context of binary classification, XGBoost models the probability of belonging to one of the two classes through a logistic function, whereas in regression, the prediction is a direct continuous estimate of the output value.

For binary classification, the prediction for a single observation $x_{i}$ is expressed as the sum of the contributions of K weak decision trees:

$$\hat{y_{i}}=\sigma \left(\sum_{k=1}^{K}f_{k}\left(x_{i}\right)\right),$$

where $\sigma \left(z\right)=\frac{1}{1+e^{-z}}$ is the sigmoid function that maps real values into the [0, 1] interval [23]. The objective of the model is to minimize the logistic loss function:

$$l\left(y_{i},\;\hat{y_{i}}\right)=-\left[y_{i}log\hat{y_{i}}+\left(1-y_{i}\right)log\;\left(1-\hat{y_{i}}\right)\right],$$

where $y_{i}\in \{0,1\}$ is the actual label of the observation. An additional regularization term Ω(f) penalizes tree complexity to avoid overfitting:

$$\Omega \left(f\right)=\gamma T+\frac{1}{2}\lambda \sum_{j=1}^{T}w_{j}^{2},$$

where *T* is the number of leaves, $w_{j}$ is the weight of leaf *j*, and *γ*, *λ* are control hyperparameters [23]. The combined objective function

$$Obj=\sum_{i=1}^{n}l\left(y_{i},\hat{y_{i}}\right)+\sum_{k=1}^{K}\Omega (f),$$

as is minimized through a boosting procedure that uses a second-order approximation via the Taylor expansion of the loss function, which allows gradients $g_{i}$ and Hessians $h_{i}$ to be calculated efficiently. These values are fundamental for the iterative construction of new trees, which are added to correct the residual errors of the current model. The choice of the best division of a node is based on the split gain, defined as

$$Gain=\frac{1}{2}\left(\frac{G_{L}^{2}}{H_{L}+\lambda }+\frac{G_{R}^{2}}{H_{R}+\lambda }-\frac{G^{2}}{H+\lambda }\right)-\gamma ,$$

where *G* and *H* are the sums of gradients and Hessians, respectively, and *L* and *R* indexes refer to left and right child nodes [23,52]. This methodology allows XGBoost to effectively balance predictive accuracy and model complexity, achieving excellent results even on high-dimensional data or data with missing values [52].

For regression, the model structure remains the same, that is, an additive sum of decision trees, but the prediction is computed directly as:

$$\hat{y_{i}}=\sum_{k=1}^{K}f_{k}(x_{i}),$$

without applying the sigmoid function. The most common loss function is the Mean Squared Error (MSE):

$$l{\left(y_{i},\hat{y_{i}}\right)}^{2}=\frac{1}{2}{\left(y_{i},\hat{y_{i}}\right)}^{2},$$

with a second-order Taylor expansion, allowing iterative updates of the model, which measures the distance between observed and predicted values. Here, the gradient is $g_{i}=\hat{y_{i}}-y_{i}$ and the Hessian is $h_{i}=1$, simplifying calculations [23]. Again, the objective is approximated

$$Obj^{\left(t\right)}\approx \sum_{i}\left[g_{i}f_{t}\left(x_{i}\right)+\frac{1}{2}h_{i}f_{t}{\left(x_{i}\right)}^{2}\right]+\Omega (f_{t})$$

Split selection and regularization are handled using the same formulas used for classification, allowing precise control of complexity and improving generalization [52].

XGBoost, in both binary classification and regression, thus exploits a gradual boosting approach where each new tree focuses on residual errors of the current model. The combined use of gradients and Hessians, along with strong regularization and computational optimizations, ensures high predictive accuracy and robustness even with complex or noisy data [23,52,53]. Its efficient memory management and parallelization make it suitable for large-scale datasets without compromising speed or model quality [53].

#### 4.2.2. Generalized Linear Model (GLM with Quasi-Poisson)

The Generalized Linear Model (GLM) with quasi-Poisson distribution extends classical linear regression to handle count data with variance greater than the mean (overdispersion). The theoretical formulation of GLMs was introduced by McCullagh and Nelder in 1989 [54] and is based on three components: a distribution from the exponential family, a link function that relates the mean of the response variable to a linear predictor, and a variance function describing how variance depends on the mean.

In the quasi-Poisson case, the distribution of data follows the assumption of a variance-mean relationship of the type:

Var(Y)=ϕμ,

where ϕ is the dispersion parameter. Unlike the standard Poisson model Var(Y)=μ, This adjustment allows handling of overdispersed data, where the variance exceeds the mean [55]. This approach is particularly suitable in clinical and epidemiological settings, where observations often derive from counts of rare and independent events that are influenced by unobserved heterogeneity, which produces higher variances.

Formally, the conditional mean $\mu_{i}\;$ of response $Y_{i}$ is linked to predictors $X_{i}$ via a logarithmic link:

$$log\;\left(\mu_{i}\right)\;=\beta_{0}+\beta_{1}X_{i1}+\beta_{2}X_{i2}+\cdots +\beta_{p}X_{ip},$$

where $\beta_{j}$ are coefficients estimated via quasi-likelihood method. Interpretation is similar to the Poisson model: a one-unit increase in predictor $X_{j}$ corresponds to a multiplicative change of $e^{\beta_{j}}$ in the mean response. The dispersion parameter ϕ corrects variance estimates, making the model more robust to overdispersion [56].

In practice, the quasi-Poisson model is used for discrete outcomes such as the number of sensitizing allergens or severity scores of allergic symptoms. This property makes it particularly suitable for studying allergic reactions, where the clinical response can be quantified in ordinal terms but treated as a count, allowing the association between the number of sensitizations and the severity of symptoms to be assessed. Moreover, unlike the standard Poisson model, it reduces the risk of underestimated variance and provides more realistic confidence intervals [57].

The use of this model fits into the broader context of GLMs, which offer a flexible framework for modelling non-Gaussian response variables, extending the predictive capabilities of linear regression and allowing complex phenomena with asymmetric distributions to be analyzed. In the field of allergology and clinical research, the application of quasi-Poisson GLM allows us to understand how sensitization to multiple allergens and their type influence the intensity of allergic reactions, providing crucial information for risk management and prevention [58].

## 5. Conclusions and Perspectives

In conclusion, this study demonstrates that nut co-sensitization in Pru p 3-sensitized patients constitutes a complex and clinically heterogeneous condition. By applying advanced artificial intelligence-based models (XGBoost and GLM), we identified distinct sensitization patterns associated with the presence and severity of allergic symptoms.

Although peanut and hazelnut sensitization was the most frequent finding in our patient cohort, peanuts are less frequently responsible for symptom onset; this is probably due to cross-reactivity and does not indicate a true allergy.

The application of AI models also allowed us to identify two subgroups of patients, the group of sensitized and symptomatic patients for peach (known primary sensitizer of LTP syndrome, which guides the onset of symptoms) and the group of asymptomatic patients for peach albeit sensitized. Machine learning has highlighted different characteristics in these two patient groups that may explain why some patients do not develop symptoms after peach ingestion. In fact, in asymptomatic people, greater sensitization to peanut and hazelnut was found. This means that in some groups of patients, nsLTP sensitization could be driven by nsLTPs of foods other than peach. These considerations currently appear to be limited and speculative. In fact, for both Aim 1 and Aim 2, the limitations of the study are represented by the absence of CRD testing on the entire patient cohort. We are not aware of the specific sensitization patterns, but we have hypothesized them based on the positive results found in SPTs or through specific IgE for whole allergens. Furthermore, with regard to Aim 1, we did not include sensitization to inhalant allergens in the study parameters, which prevents us from obtaining a broader picture of sensitization. Finally, the findings of Aim 3 would also be reinforced by a better understanding of sensitization patterns through CRD in order to better achieve risk stratification. However, these results provide a starting point for further studies on the different sensitization patterns in LTP syndrome.

Finally, walnut sensitization emerged as the strongest predictor of severe clinical reactions, followed by peanut, hazelnut, and almond. Moreover, an increasing number of sensitizations correlated with higher clinical severity, supporting a cumulative immunologic effect within the LTP syndrome spectrum.

The integration of AI-driven predictive modelling with molecular allergy diagnostics represents a valuable approach for improving risk stratification and personalized patient management. These results provide novel insights into nsLTP-related cross-reactivity mechanisms and could contribute to the development of more precise diagnostic and preventive strategies in food allergy.

## Figures and Tables

**Table 1:** Prognostic factors in nsLTPs allergy.

Protective Cofactors	Prognostically Negative Cofactors
Co-sensitization to profilins	Mono sensitization to nsLTPs
Co-sensitization to PR-10	Ingestion of food on an empty stomach
	Physical exercise (FDEIA)
	Intake of NSAIDs and PPI
	Alcohol Ingestion
	Hormonal factors

**Table 2:** Distribution of positivity to different nuts in 109 patients sensitized to Pru p 3 with symptoms to nuts.

Tree Nuts	Sensitized Patients	Percentage (%)
PEANUT	95	87.2%
HAZELNUT	91	83.5%
ALMOND	64	58.7%
WALNUT	43	39.4%

**Table 3:** Distribution of positivity to nuts in 104 patients sensitized to Pru p 3 and symptomatic for peach.

Tree Nuts	Sensitized Patients	Percentage (%)
PEANUT	89	85.6%
HAZELNUT	86	82.7%
ALMOND	54	51.9%
WALNUT	38	36.5%

**Table 4:** Predictive importance (Gain, Cover, Frequency) and positive clinical concordance of nuts in the XGBoost model for predicting the presence of clinical symptoms.

	Feature	Gain	Cover	Frequency	% Positive Agreement
1	HAZELNUT_NUM	0.34093962	0.46432531	0.30496454	96.6
2	WALNUT_NUM	0.31263345	0.21451735	0.27304965	96.2
3	ALMOND_NUM	0.29980353	0.26232746	0.37943262	95.2
4	PEANUT_NUM	0.04662341	0.05882988	0.04255319	94.4

**Table 5:** Positive test counts and clinical concordance for tree nut allergens.

Allergen	Total Positive Tests	Concordant Symptoms	% Positive Agreement
PEANUT_NUM	124	117	94.4
ALMOND_NUM	83	79	95.2
HAZELNUT_NUM	118	114	96.6
WALNUT_NUM	53	51	96.2

**Table 6:** Confusion matrix and performance indices for the prediction of clinical symptom absence (Positive class: 0).

		Reference 0	Reference 1
Confusion matrix	Predicted 0	0	0
	Predicted 1	2	26
Summary indices	Accuracy	0.9286 (95% CI: 0.765–0.9912)	
	No Information Rate	0.9286	
	*p*-value (Acc > NIR)	0.6768	
	Kappa	0.00	
	McNemar’s test *p*-value	0.4795	
Classification indices (positive class = 0)	Sensitivity	0.00	
	Specificity	1.00	
	Positive predictive value	Not calculable (no true positives)	
	Negative predictive value	0.92857	
	Prevalence	0.07143	
	Balanced accuracy	0.50	

**Table 7:** Distribution of positivity for nuts in 34 patients asymptomatic for peach but symptomatic for nuts.

Tree Nuts	Sensitized Patients	Percentage (%)
PEANUT	28	82.4%
HAZELNUT	28	82.4%
ALMOND	25	73.5%
WALNUT	13	38.2%

**Table 8:** Comparison between the nuts most frequently positive in symptomatic patients for peach vs. asymptomatic patients for peach.

	Feature	Gain	Cover	Frequency
1	ALMOND_NUM	0.3136063	0.2913558	0.3719512
2	WALNUT_NUM	0.3065781	0.2516025	0.2573171
3	HAZELNUT_NUM	0.2600354	0.2712170	0.2451220
4	PEANUT_NUM	0.1197801	0.1858246	0.1256098

**Table 9:** Confusion matrix and performance indices for the prediction of specific symptoms in peach (Positive class: 1).

		Reference 0	Reference 1
Confusion matrix	Prediction 0	1	3
	Prediction 1	6	18
Summary indices	Accuracy	0.6786 (95% CI: 0.4765–0.8412)	
	No Information Rate	0.7500	
	*p*-value (Acc > NIR)	0.8615	
	Kappa	0.0000	
	McNemar’s test *p*-value	0.5050	
Classification indices (positive class = 1)	Sensitivity	0.8571	
	Specificity	0.1429	
	Positive predictive value	0.7500	
	Negative predictive value	0.2500	
	Prevalence	0.7500	
	Detection rate	0.6429	
	Detection prevalence	0.8571	
	Balanced accuracy	0.5000	

**Table 10:** Percentage of symptomatic vs. asymptomatic for peach among patients sensitized to Pru p 3.

	PEACH_POS	SYM_PEACH_LABEL	N	Total	Percentage (%)
1	Sensitized	Symptomatic	104	145	71.7%
2	Sensitized	Asymptomatic	41	145	28.3%

**Table 11:** Importance of predictive variables in the XGBoost model for the clinical severity of allergic reactions (scale 0–4), (n = 109).

	Feature	Gain	Cover	Frequency	Importance
1	WALNUT_NUM	0.3348775	0.2521686	0.2438272	0.3348775
2	PEANUT_NUM	0.2632098	0.2321930	0.1898148	0.2632098
3	HAZELNUT_NUM	0.2114242	0.2633476	0.2114198	0.2114242
4	ALMOND_NUM	0.1904885	0.2522908	0.3549383	0.1904885

**Table 12:** Mapping of positive tree nut tests to clinical severity scores (0–4).

Tree Nut	Test Result	Severity	N. Patients	Total	Percentage (%)
Peanut	Positive	0	0	94	0.0
Peanut	Positive	1	0	94	0.0
Peanut	Positive	2	73	94	77.7
Peanut	Positive	3	7	94	7.4
Peanut	Positive	4	14	94	14.9
Hazelnut	Positive	0	0	89	0.0
Hazelnut	Positive	1	0	89	0.0
Hazelnut	Positive	2	65	89	73.0
Hazelnut	Positive	3	8	89	9.0
Hazelnut	Positive	4	16	89	18.0
Almond	Positive	0	0	63	0.0
Almond	Positive	1	0	63	0.0
Almond	Positive	2	46	63	73.0
Almond	Positive	3	6	63	9.5
Almond	Positive	4	11	63	17.5
Walnut	Positive	0	0	43	0.0
Walnut	Positive	1	0	43	0.0
Walnut	Positive	2	30	43	69.8
Walnut	Positive	3	3	43	7.0
Walnut	Positive	4	10	43	23.3

**Table 13:** Regression model (GLM) with quasi-Poisson distribution for predicting clinical severity (SEVERITY) based on the number of positive food allergens (NUM_ALLERGIES).

Call:				
glm (formula = SEVERITY ~ NUM_ALLERGIES, family = quasipoisson(), data = df_109)				
Coefficients:				
	Estimate	Std. Error	t value	Pr (>|t|)
(Intercept)	0.67815	0.11558	5.867	5.01 × 10^−8^ ***
NUM_ALLERGIES	0.05149	0.02989	1.722	0.0879

Signif. codes: 0 ‘***’ 0.001 ‘**’ 0.01 ‘*’ 0.05 ‘.’ 0.1 ‘ ’ 1				
(Dispersion parameter for quasipoisson family taken to be 0.2257745)				
Null deviance: 22.113 on 108 degrees of freedom				
Residual deviance: 21.440 on 107 degrees of freedom				
AIC: NA				
Number of Fisher Scoring iterations: 4				

## Data Availability

The original contributions presented in the study are included in the article/Supplementary Materials. Further inquiries can be directed to the corresponding author.

## Supplementary Materials

The supporting information can be downloaded at: https://www.mdpi.com/article/10.3390/ijms27031223/s1.

## Abbreviations

The following abbreviations are used in this manuscript:

nsLTPs	non-specific lipid transfer proteins
SPT	Skin prick test
PA	Peach allergic
PT	Peach tolerant
NA	Nuts allergic
NT	Nuts tolerant
GLM	Generalized Linear Model
OAS	Oral allergic syndrome
