# Supplementary material for: The Role of Nut Sensitization in Pru p 3-Sensitized Patients: A XGBoost and Generalized Linear Model Application
Source: Int J Mol Sci. 2026 Jan 26;27(3):1223. doi: 10.3390/ijms27031223 (PMC12897624; doi:10.3390/ijms27031223)
Supplement: Supplementary file 1 [file ijms-27-01223-s001.zip › ijms-3991463-supplementary.pdf]

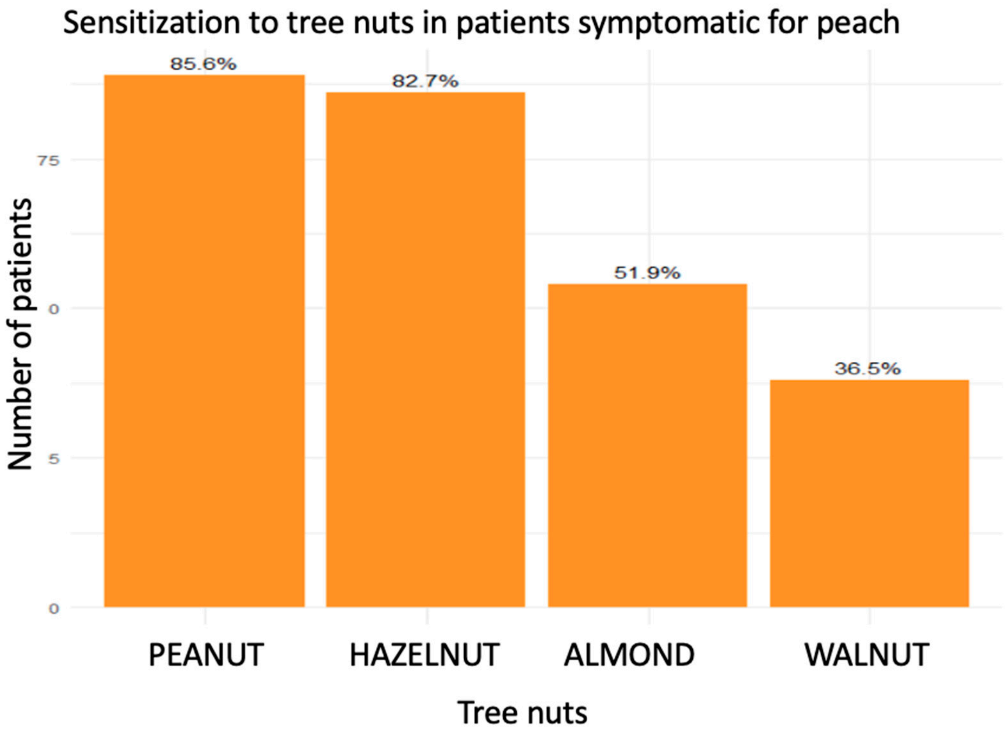

Figure S1: Relative importance of nuts in predicting the presence of clinical symptoms (XGBoost model)

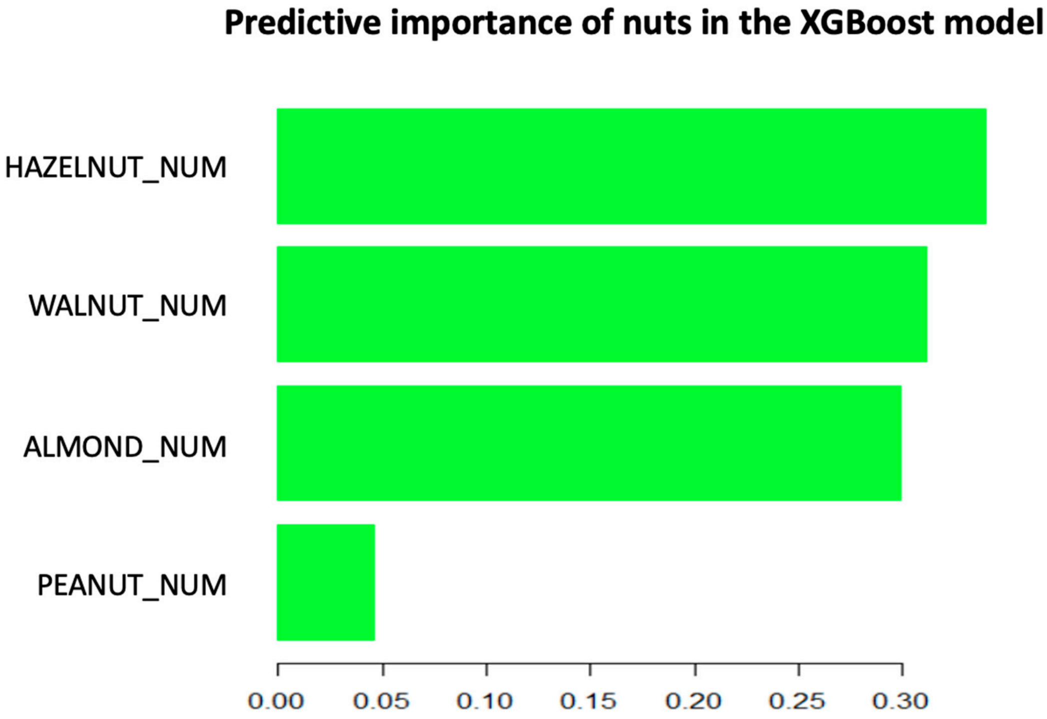

Figure S2. Predictive importance of nuts in the XGBoost model

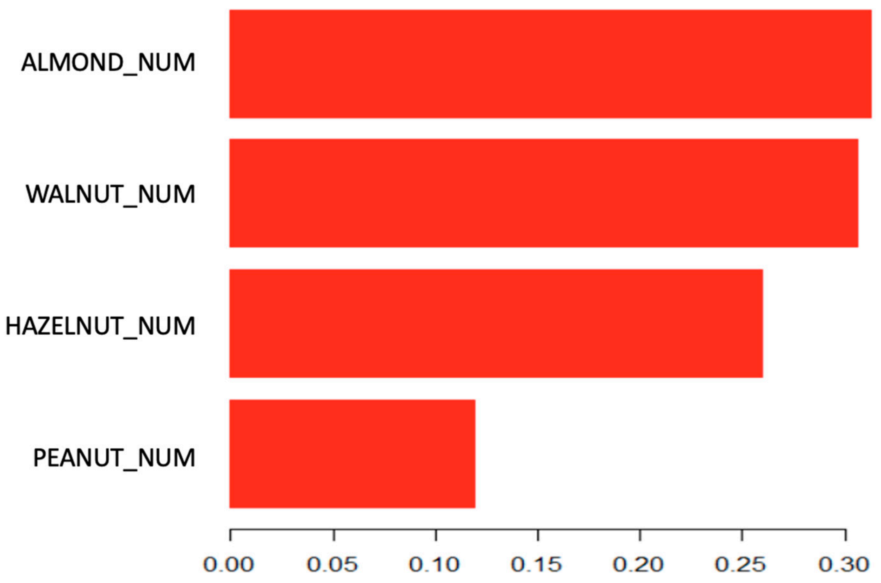

Figure S3. Importance of food allergens in predicting the presence of symptoms with peach (XGBoost model)

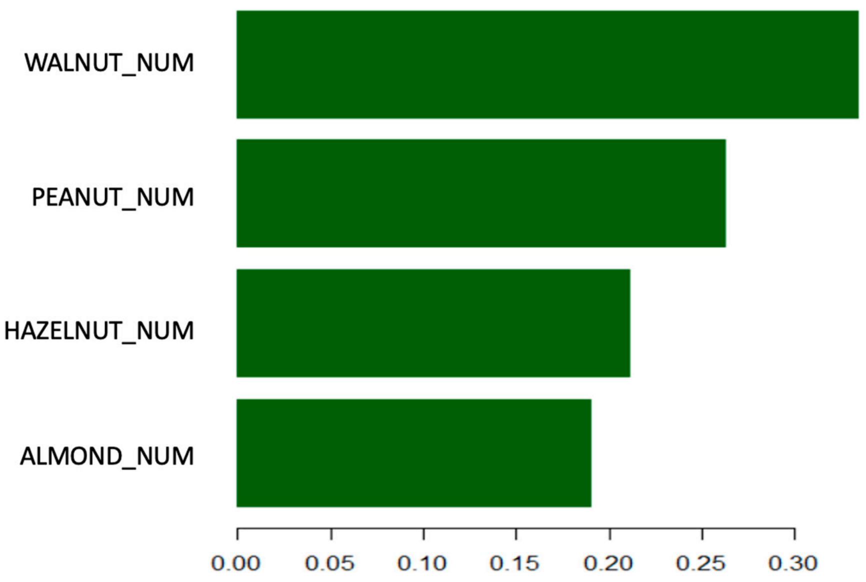

Figure S4. Importance of variables in the XGBoost model for the clinical severity of allergic reactions

The trend illustrated in the figure shows how the influence of walnuts clearly exceeds that of other allergens, contributing more significantly to the risk profile.
